# The impact of participatory teaching methods on medical students’ perception of their abilities and knowledge of epidemiology and statistics

**DOI:** 10.1371/journal.pone.0202769

**Published:** 2018-08-22

**Authors:** Margarita Rubio, María Sánchez-Ronco, Rosa Mohedano, Asunción Hernando

**Affiliations:** School of Biomedical Sciences and Health, Universidad Europea de Madrid, Madrid, Spain; University Lyon 1 Faculty of Dental Medicine, FRANCE

## Abstract

Statistics and Epidemiology are crucial both in clinical decision-making and clinical research. Teaching these disciplines in a Bachelor’s Degree in Medicine is a significant challenge. In this paper, we aim to describe two participatory teaching methods used in a yearlong second-year course that includes both Epidemiology and Statistics, and to analyze how these two methodologies affect the students’ perception of the course and their abilities related to these subjects. Both methodologies consist in carrying out a specific practical activity. The first practical activity is carried out using a website and aims to help students understand concepts and interpret information; the second involves analyzing a database using a statistical package and, subsequently, producing a scientific report. In addition, we prepared a questionnaire to find out the students’ perception of these issues. The nine questionnaire items were assessed using a rating scale and adapted to characteristics of the course, which covers Epidemiology and Statistics in an integrated manner. Then we assessed the differences in perception before and after the activities were carried out. The results show that the students’ perception improved significantly in the following items: “importance of Statistics and Epidemiology in Medicine”; “usefulness in clinical practice”; “understanding concepts”; “ability to perform a statistical analysis”; and “ability to sort data”. The difference was not significant in the remaining four items. In conclusion, the students’ perception of their ability in Statistics and Epidemiology significantly improved after completing the practical activities, and their perception of importance and usefulness of these subjects also improved.

## Introduction

In all areas of Medicine, professionals consider that Biostatistics and Epidemiology are fundamental tools for medical research and clinical decision-making, but it is still difficult to ensure that undergraduates acquire the necessary skills in those subjects [1, 2]. Some authors have argued that this teaching should focus exclusively on the concepts [3], while others have proposed a balance between skills related to calculations and those related to reasoning and understanding concepts [4, 5].

Some studies have analyzed the opinion of Medicine graduates, who seem to think that statistics is an important but very complicated subject, recognizing their difficulties in understanding the terminology used and the statistical methods that are appropriate for each type of study. Because of these deficiencies in their knowledge of Biostatistics, almost half of the respondents did not use statistics adequately when conducting their research, which negatively affected its quality [1]. Lack of knowledge of statistics can lead to errors in the selection and size of the sample, in the choice and interpretation of statistical tests for analysis. All this may lead to erroneous conclusions.

In the past, attention was given mainly to improving the cognitive aspects of teaching Statistics, whereas more recently, research has focused on non-cognitive aspects such as the attitude of students towards the subject. This is a multidimensional approach that includes affective, cognitive and behavioral components [6]. Several studies show that students’ perception of the subject and their attitude towards it are important when it comes to achieving good learning outcomes [7, 8].

A recently published meta-analysis [9] states that medical students generally have a positive attitude towards statistics, but different studies offer different data regarding how their perception of their cognitive skills, interest and difficulties change after finishing the courses. The conclusion was that both actual math skills and attitude to the subject influence the students’ performance in Statistics courses, and that it is important to also improve the students’ perception of their own skills.

In a study using focus groups to explore the students’ view of Biostatistics, students recognized the need to receive training in the early years of the degree, highlighting their lack of confidence in the subject and perception of insufficient support received during their training. They considered it very important to work with scientific articles and virtual environments and most felt that the basic concepts should be explained in person in small groups that allow interaction between students and teacher; they considered online materials complementary. The participants gave great importance to the methodologies that allow them to put content into practice [10].

Given the challenges posed by new teaching methodologies, it has begun to be considered important and even necessary for research to go beyond theoretical reflections and focus on describing the design of pedagogical strategies and specific teaching methods that use virtual environments and technological tools at different educational levels. Rather than just use the technologies, it is essential to plan both face-to-face and virtual activities adapted to the context [11–13]. With this article, we wanted to share our teaching experience in a course that requires teaching both Biostatistics and Epidemiology, which in turn involves using participatory methodologies that incorporate technological tools.

Following implementation of the Basic Epidemiology and Applied Biostatistics course, in the second year of the Bachelor’s Degree in Medicine, we conducted this study with the following objectives:

- To describe two participatory methodologies, each consisting of a practical activity designed to ensure better understanding and application of concepts.
- To analyze the correlation between the grades that students obtain in the theory exams and the results obtained in the practical activities.
- To analyze the effect of these practical activities on students’ perception of their abilities and knowledge of Epidemiology and Statistics.

## Method

The practical activities described form part of the "Basic Epidemiology and Applied Biostatistics" course delivered in the second year of the Bachelor’s Degree in Medicine of a Spanish university.

### Description of the course

In the context of the "Basic Epidemiology and Applied Biostatistics" course delivered in the second year of the Bachelor’s Degree in Medicine, the main objective of the theoretical content, the practical activities, and the teaching methodologies used is to transmit an integrated view of Epidemiology and Biostatistics. To this end, Statistics is presented to the student throughout the course as a tool that allows information to be analyzed and conclusions to be reached using a scientific methodology. Although the student should learn to perform and interpret some statistical analyses, we consider it more important that he/she be able to understand the statistical methodology most frequently used in medical studies, correctly interpret the results obtained, and acquire the knowledge necessary for developing an adequate critical spirit for interpreting medical information. Table 1 shows the course’s learning objectives and the activities to be carried out.

**Table 1:** Training design: Objectives and activities of the course.

Week	Activities (A)	Learning objectives (O)
1–3	A1. Lecture A2. Working in pairs with summaries of scientific articles A3. Self-assessment activities aimed at applying the concepts	O1. Understand the concept of health and its relationship with the determinants of health O2. Understand the concept of Epidemiology and its importance in the practice of Medicine
4	A1. Lecture A4. Finding indicator information in demographic applications A3. Self-assessment activities aimed at applying the concepts	O3. Interpret the basic aspects of demography and their usefulness in public health
5–7	A1. Lecture A5. Identifying epidemiological studies A6. Designing a study A3. Self-assessment activities aimed at applying the concepts	O4. Understand the epidemiological method O5. Compare the different types of epidemiological studies, their characteristics and their application in research
8–21	A1. Lecture A7. Problem solving A3. Self-assessment activities aimed at applying the concepts A8. Epidemiological simulation (Epiville during weeks 19–21)	O6 Understand the relationship between probability and clinical reasoning processes O7. Understand the concepts of association and risk in epidemiology O8. Apply probability to the evaluation of diagnostic tests O9. Apply probability distributions and understand their usefulness in solving epidemiological problems O10. Interpret the descriptive analysis of qualitative and quantitative variables O11. Understand the usefulness of inferential epidemiology O12. Perform and interpret statistical estimations of qualitative and quantitative parameters O13. Understand the bivariate and multivariate statistical tests to perform a hypothesis test O14. Apply the hypothesis testing to epidemiological studies
22–25	A9. Using software for statistical analysis activities (EpiInfo) A10. Presenting the results of an analysis in article format	O15. Know how a statistical program works O16. Perform descriptive and analytical statistics of a database O17. Interpret and write up the results of a statistical analysis using the format of a scientific article
26–27	A1. Lecture A11. Bibliographic sessions	O18. Assess whether the statistical tests used in an epidemiological study are suited to its objectives O19. Interpret the results of the analysis conducted in a study O20. Understand the quality criteria of scientific studies O21. Apply the quality criteria to published studies
28–34	A1. Lecture A12. Group work on a chronic disease A13. Group tutorials A14. Oral presentation of the work	O22. Understand the epidemiology of communicable diseases and their prevention O23. Understand the epidemiology of the most prevalent chronic diseases, their risk factors and the strategies for their prevention

The content corresponding to biostatistics, bases of epidemiology, and epidemiology of communicable diseases is carried out through the following methodologies or activities: lectures, problem solving and case studies, software use practical activities for statistical analyses, practical activities involving simulation of epidemiological problems, critical analysis of a scientific article, and exposition of the article in a bibliographical session.

The content corresponding to epidemiology of non-communicable diseases is carried out through supervised preparation and presentation of a monographic assignment. This assignment involves carrying out a bibliographic search and selecting the best information available. The student is faced with a complex bibliographic search for the first time and therefore requires several advisory sessions to guide him/her through the process of finding and analyzing the information. The students carry out this assignment in groups of three or four, so that they also develop the ability to work as a team. Furthermore, this distribution in groups means that the number of advisory sessions and presentations does not render the logistics of the activity nonviable when the groups are made up of a large number of students.

Throughout the course, different activities are carried out in the classroom to apply the knowledge acquired to practical cases that mirror common situations in the field of Medicine. At the end of this process, two practical activities that integrate the most significant aspects of the course are carried out. The aim is to improve the skills and competencies that are considered fundamental in a medical student and that are the objectives of the course.

The first of these practical activities is a multimedia simulation activity based on the Columbia University website "Epiville" [14].

This tool is a collaborative project between the Department of Epidemiology of the School of Public Health and Columbia University’s Center for New Media Teaching and Learning. It was developed to provide a better learning environment that would allow students to more efficiently master the fundamental principles of Epidemiology: causal inference, cohort study, case control study and bias. The website consists of independent modules in which the student will investigate a number of public health problems, assuming the role of an intern at the Department of Health in the fictitious town of Epiville. Each module (causal inference, cohort study, case control study, and bias) is divided into the following sections: Introduction, Learning Objectives, Student Role, Study Design, Data Collection, Data Analysis, and Discussion Questions. The Introduction, Learning Objectives, Student Role and Study Design sections provide all the necessary information on the public health problem and the study model selected for its investigation. The student will start by collecting relevant data (Data Collection), including television and radio reports, informative materials from the Epiville Health Department, and interviews with local residents. Then the student will use all the gathered information to address key theoretical and analytical aspects, simulating a practical experience of applying epidemiological methods (Data Analysis, and Discussion Questions). In each module, the students can find links to documents containing further information on ethical and administrative aspects of the studies, a glossary of epidemiological terms, and interactive multiple choice questions with reasoned solutions. This practical activity involves six two-hour sessions in which the students work in pairs under the guidance of a course professor to whom they must present a report on each completed module. At the end of each module, students have to read and analyze a scientific article, which will be related to the topic worked on in that module, and then answer a series of questions about the content of the article aimed at checking their understanding of methodological concepts and statistical data. The aim is for students to make connections between the simulated situation and the actual published studies.

The second practical activity involves carrying out a statistical analysis of an anonymized clinical database, and the subsequent interpretation and writing of a report on the results of the analysis, simulating the structure of the "Results" section of a scientific article. For this purpose, the student has a Microsoft Excel database with information on 21 variables of 316 patients and the statistical program EpiInfo, developed by the Centers for Disease Control and Prevention (CDC) [15]. The practical activity takes place over 10 two-hour sessions, with students working in pairs. All sessions are held in the presence of a professor who guides them and answers their queries. In the first session, the professor informs the students about the problem to be studied, clarifies doubts about the variables included, explains in detail what the data was collected for, and explains the work they have to do using that database. Students are provided with a detailed guide to the activity, specifying the purpose of the study, the analyses they have to carry out, and instructions for using the program. They should describe the characteristics of the patients included in the study by calculating the frequencies of the categorical variables (absolute number, percentage, and 95% confidence interval), and by using central tendency and dispersion measurements for the quantitative variables. They must establish hypotheses and perform bivariate analysis using parametric or nonparametric tests that are appropriate to each case. Finally, they must perform the hypotheses testing and thereby determine whether there is a statistical association between the variables studied.

The processing and analysis they perform should be recorded in a document that simulates the "Results" and "Conclusions" sections of a scientific article. To do this, they should organize the information into text, tables and graphs, paying attention to previously published articles on the same subject in order to both write and organize the information in a suitable manner. The final document is delivered to the professor, who will grade it while also taking into account the students’ attitude during the sessions.

### Questionnaire design and study phases

**Fig 1:**
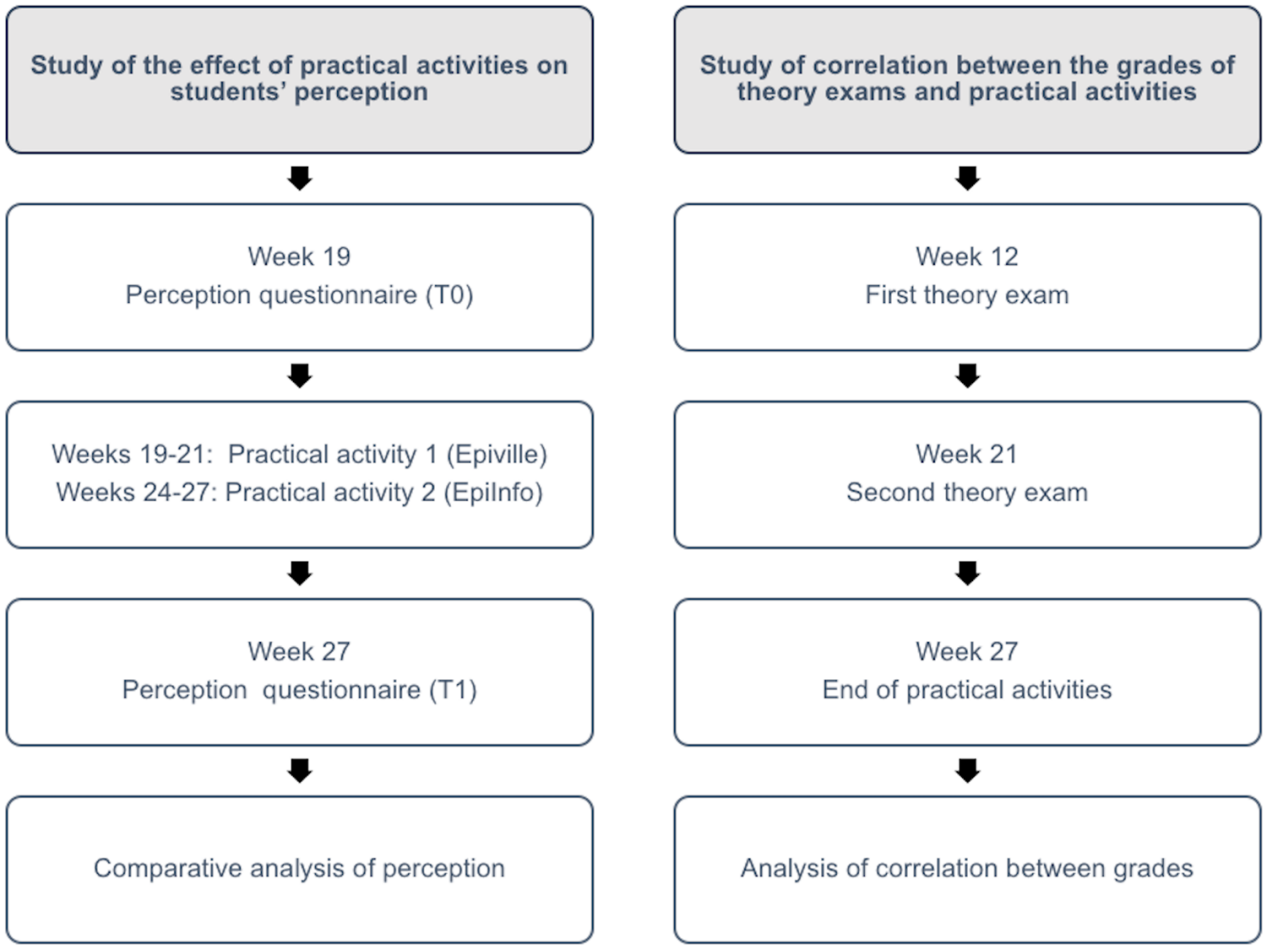
Study phases.

Ethical approval for this study was obtained from Universidad Europea de Madrid’s Ethics Committee. The phases of the study are shown in Fig 1.

All students who participated in the study signed an informed consent, which explained the aims of the study, guaranteed anonymity and explicitly declared their consent for the results to be published.

The questionnaires available in the bibliography for measuring students’ perception of Statistics focus specifically on courses that contain only Statistics content and do not include aspects related to Epidemiology. Given that the integration of Statistics and Epidemiology entails a different approach and different content, the course professors prepared a questionnaire adapted to the distinctive features of the course to find out students’ perception of the course and their skills in relation its content. The questionnaire was reviewed by five experts in research methodology.

The questionnaire gathers information about the students’ perception of two aspects: first, the importance and usefulness of the course (four items); and second, the students’ ability to carry out the activities related to the course’s most important objectives (five items). The questionnaire consists of nine items (Tables 2 and 3) with four response options that follow a rating scale ranged from 1 to 4, with 1 being “Strongly disagree” and 4 “Strongly agree”. The first item refers to the importance of Statistics and Epidemiology in Medicine, the second to their usefulness for the professional practice of Medicine, the third to the relationship between Epidemiology and Statistics, and the fourth to the understanding of the main concepts of Epidemiology and Biostatistics. The rest of the items assess the students’ perception of their ability to: understand concepts, read and understand scientific articles, perform a statistical analysis, sort data, write up the results of an analysis, and interpret the results of statistical tests.

**Table 2:** Students’ perception of the importance and usefulness of the subject. Number and percentage of students who answered 3 or 4, before and after completing the practical activities.

	Before n = 120	After n = 135
Indicate your degree of agreement with the following statements:		
Epidemiology and Biostatistics are important for Medicine as a science	116	131
	97,5%	97,8%
I believe that the knowledge and skills I have acquired in this course are fundamental for my professional performance in Medicine	103	120
	85,8%	90,2%
I understand the relationship between Epidemiology and Biostatistics	112	130
	94,1%	96,3%
I understand the main concepts of Epidemiology and Biostatistics	114	131
	96,6%	97,8%

**Table 3:** Students’ perception of their skills for different tasks related to the course content. Number and percentage of students who answered 3 or 4, before and after completing the practical activities.

	Before n = 120	After n = 135
Rate from 1 to 4 your ability to:		
Read and understand scientific articles	99	119
	82,5%	88,8%
Perform a basic statistical analysis	72	107
	60,0%	80,5%
Sort the data of a statistical analysis	83	115
	69,7%	85,8%
Write the results of a statistical analysis	85	113
	70,8%	83,7%
Interpret the results of the statistical tests	99	122
	82,5%	90,4%

The questionnaire was made available through the Blackboard Learn platform using the "Surveys" option that guarantees anonymity. The students were informed of the development and objectives of the study and encouraged to participate in it during the classes prior to completion of the first questionnaire. The students answered the questionnaire during the first 15 minutes of class on two occasions: the day on which the practical activities began (six months after the start of the course) and the day on which the activities ended (16 weeks later). Participation in the study was voluntary.

During the first semester, before the practical activities, the students did two theory exams including questions about theoretical content and its applications. Carrying out the practical activities (simulation and statistical analysis) should serve to reinforce this content and improve the students’ hands-on skills. Each theory exam consisted of a test with 40 multiple-choice questions and a single correct answer, and five open questions. The final grade of the exam was obtained by calculating the weighted average of the multiple-choice test (40%) and the open-ended questions (60%). The first exam included questions on learning objectives O1 to O8 (see Table 1), and the second exam comprised questions on objectives O9 to O14. We studied the correlation between the theory exam grades and those of the practical activities. We excluded from this analysis the 10 students who did not take these theory exams or do the practical activities (n = 164).

### Statistical analysis

Frequencies were expressed as absolute number and percentage. The scores on the rating scale were shown as mean ± standard deviation. To compare the scores obtained, Student’s t-test for independent samples was used after analysis of variance homogeneity using the Levene test.

To analyze the possible correlation between the grades of the course’s theory exams and those of the practical activities, we calculated the arithmetic mean of the two theory exams and the arithmetic mean of the grades obtained in the practical activities. This analysis included all the students enrolled in the course, with the exception of 14 students who did not completely finish the practical activities and, therefore, did not have a grade. For the correlation analysis, a Pearson correlation was performed after checking the normality of grade distributions using the Kolmogorov-Smirnov test). We considered statistically significant *P* values of less than 0.05. IBM SPSS Statistics version 21.0 (IBM, Armonk, New York, USA) was used for data processing.

## Results

### Perception results

In academic year 2016/17, 174 students (72.4% female and all of them Caucasian) enrolled in the course and all of them were asked to participate in the research after the objective of the study was explained to them. Before and after the two practical activities described, the students who participated in the study completed the perception questionnaires. A total of 120 students voluntarily agreed to participate and completed the questionnaires on the day they began the practical activities (response rate: 69%), and 135 students did so after finishing these activities (response rate: 77.6%).

As shown in Table 2, the vast majority of students agreed with the statements relating to the importance and usefulness of the course and their understanding of it, both before and after completing the practical activities. For all the statements, there is an increase in the percentage of students who answer 3 ("agree") or 4 ("strongly agree") after carrying out the activities. Table 4 shows the comparison of average scores for these items. The increase in the average score is statistically significant in items 1 ("Epidemiology and Biostatistics are important for Medicine as a science"), 2 ("I believe that the knowledge and competencies that I have acquired in this course are fundamental for my professional performance in the field of Medicine"), and 4 ("I understand the main concepts of Epidemiology and Biostatistics").

**Table 4:** Average value response (mean ± standard deviation), before and after completing the practical activities.

	Before n = 120	After n = 135	p
Indicate your degree of agreement with the following statements (1-strongly disagree; 4-strongly agree)			
1.- Epidemiology and Biostatistics are important for Medicine as a science	3,44 ± 0,58	3,60 ± 0,54	0,02
2.- I believe that the knowledge and skills I have acquired in this course are fundamental for my professional performance in Medicine	3,02 ± 0,55	3,17 ± 0,58	0,03
3.- I understand the relationship between Epidemiology and Biostatistics	3,26 ± 0,56	3,40 ± 0,59	0,05
4.- I understand the main concepts of Epidemiology and Biostatistics	3,17 ± 0,46	3,31 ± 0,51	0,03
Rate from 1 to 4 your ability to:			
5.- Read and understand scientific articles	2,94 ± 0,54	3,04 ± 0,55	0,13
6.- Perform a basic statistical analysis	2,68 ± 0,69	2,95 ± 0,58	0,001
7.- Sort the data of a statistical analysis	2,86 ± 0,67	3,07 ± 0,59	0,01
8.- Write the results of a statistical analysis	2,88 ± 0,69	3,04 ± 0,63	0,05
9.- Interpret the results of the statistical tests	3,03 ± 0,62	3,17 ± 0,60	0,08

Regarding the statements about the students’ perception of their skills for different tasks related to the course content, the majority answered "3" or "4" both before and after the practical activities (Table 3), and this percentage increased after the activities had been carried out. Table 4 shows the average scores (items 5 to 9). The lowest score before performing the practical activities was for item 6 ("Assess your ability to perform a basic statistical analysis") with an average of 2.68 ± 0.69 points, followed by item 7 ("Sort the data of a statistical analysis") with an average of 2.86 ± 0.67. The scores for these two items increased significantly, after the practical activities had been carried out (Table 4).

### Correlation between theory exams and practical activities grades

We studied the correlation between the theory exam grades and those of the practical activities (n = 164). The overall average grade for the theory exams was 6.8 ± 1.3 and the average grade for the practical activities was 8.1 ± 0.8 out of 10. The correlation analysis showed that there was a significant and positive correlation between the mean of the grades of the theory exams and those of the practical activities (p <0.001, r = 0.4).

## Discussion

Teaching Statistics and Epidemiology in the early years of the Bachelor’s Degree in Medicine is an important but complicated task. It is the first step in getting medical students to acquire skills related to statistics, to understand scientific articles and assess their quality, and to apply the best scientific evidence to their clinical practice. This study has allowed us to explore a learning experience that integrates, in the Bachelor’s Degree in Medicine, Biostatistics and Epidemiology. Over six years of experience, the methodologies used have gradually improved, and increasing importance has been attached to those that allow students to apply theoretical knowledge to situations resembling those of clinical practice. For this reason, we set out to evaluate the effect of the practical activities on the students’ perception of the course and of the skills acquired. The first practical activity centered on concepts and used a website with epidemiological simulation content, and the second focused on calculations and statistical analysis using software, the aim being to balancing these two aspects (concepts and calculations) that are considered fundamental in this area of learning.

Our questionnaire explored students’ perception of the basic skills they should develop during the course, and on completing the practical activities, between 80 and 90% of them felt they were able to understand a scientific article, and to perform and interpret a basic statistical analysis. The practical activities improved the students’ perception of their abilities in all the explored competencies, this change being statistically significant in the following aspects: understanding of concepts, performing statistical analysis, and organizing data.

Six months after the beginning of the course, but before using the participatory methodologies, 97.5% of our students considered that Epidemiology and Biostatistics are important in Medicine, and 87.8% thought that what they learned in the course would serve them as practicing professionals in the future. The students’ perception at this moment refers to their experience in these first six months, and the fact that the scores are favorable clearly suggests that the students already understand at this point the importance of the course. The average score of these two items improved significantly, after they had finished the practical activities. Studies assessing the importance of Statistics (without being combined with Epidemiology) obtain lower scores [9], which suggests that integrating Epidemiology in the Statistics learning process can improve students’ perception, since it allows the teaching of statistical skills and knowledge to be geared towards medical practice. Our data are similar to those provided by Gore et al. [1], in whose study 98.4% of the respondents agreed that biostatistics is important for research. This study [1] includes 76% of graduates and 24% of final-year students, so it can be considered that the experience makes them more aware of the importance of statistics.

We have not found any publications that describe students’ perception of the website we use in the course [14] (http://epiville.ccnmtl.columbia.edu/), although there is some research on the use of different websites [16, 17] and "blended" environments [18] in Medicine degree studies. Smucny and Epling [16] used a website to develop diagnostic reasoning and the students rated it better than other activities carried out with the same objective. Bernardo et al. [17] developed and tested a five-week online course on experimental surgery for medical students that included lectures, videos and collaborative activities and obtained good results in terms of knowledge acquisition and the students’ perception of the activity. Milic et al. [18] compared the results obtained by the Medicine students in the Statistics course, using in one group a totally face-to-face teaching methodology and in another group, a methodology combining classroom hours with online teaching that included the use of websites and other multimedia materials. The grades obtained in both statistics and theoretical knowledge were similar in both groups when the analysis was adjusted for different factors such as time spent studying or the students’ grades in their undergraduate studies. Those with better grades in their studies preferred the "blended" format. The authors conclude that this type of methodology can be an efficient and attractive alternative to the traditional class for this type of subject.

As professors in these areas of knowledge, we are aware of the importance of participatory methodologies and new technologies in helping students to improve their skills, but they can also serve to improve the students’ perception of these skills, an aspect that may be linked to achieving better learning outcomes [7, 9].

A study that included more than 800 students from different areas of knowledge analyzed their perception of the didactic website used in their courses [12], with 77.8% of the students saying they "agree" or "totally agree" with its usefulness for developing their knowledge. Furthermore, 89% of the participants expressed a high degree of satisfaction with the use of the websites, with no differences found in terms of the age or gender of the participants. After the analyses carried out, the authors consider that these tools can contribute positively in the configuration of the students’ learning processes.

A recent study conducted in the Bachelor’s Degree in Pedagogy described the students’ assessment of the usefulness of various activities carried out in courses with methodological content, including Applied Statistics [19]. The results indicate that students perceive that the activities involving greater involvement and collaboration with other students are those that help them achieve meaningful learning. Our experience reaffirms these results, since the participatory methodologies described in this study require the student’s direct involvement in the activity. Moreover, the activity is not performed individually and therefore requires that students work together, the entire process being carried out, and the final result achieved, in collaboration with a fellow student. To accomplish this goal, the professor is present in all sessions to resolve any doubts the students may have, and also to make sure that all students get involved in the activity. We believe that this way of working also allows students to feel more secure, an aspect that can help improve their attitude to learning. In relation to these aspects, Milic et al. [9] found an interesting correlation between cognitive skills and attitude in their meta-analysis.

Using computer programs for data analysis in Biostatistics courses is common practice in medical schools, but we have found only one study that evaluates the EpiInfo package as a teaching tool [20]. In this study, conducted in Italy and featuring the participation of 300 health workers and medical students, the authors conclude that using EpiInfo in the teaching of Statistics and Epidemiology facilitates the understanding of concepts and allows researchers to more easily perform their clinical-epidemiological investigations. In our experience, this software is freely accessible, endorsed by a scientific institution of recognized international prestige, easy to use, three aspects of great importance for enabling students to become familiar with the process involved in carrying out a statistical analysis. There are other programs or statistical packages that are frequently used both in teaching and in research, but which, in our opinion, do not possess these characteristics of accessibility and simplicity of use.

The participants in the comparative study of perception were the students who attended class that day and agreed to participate in the study anonymously. For this reason, the response rate is different on the two occasions when the questionnaire was completed and we could not pair the results.

In our study, we found a significant and positive correlation between the grades of the theory exams and those of the practical activities. In our opinion, this result indicates that the course is constructed in such a way that there is a connection between the theoretical and practical content taught in the classes and the competencies that are developed through the practical activities. However, the correlation coefficient was moderate-low (r = 0.4), which could be due to the fact they measure different aspects of learning. Theory tests measure theoretical knowledge while practical activities measure the ability to apply this knowledge in practice through simulation and statistical analysis.

## Conclusions

The analysis of the results obtained in our study allows us to reach the following conclusions:

- Our Medicine undergraduates perceive that integrated knowledge of Epidemiology and Statistics is important and useful for medical practice, and this perception improves after completion of the practical activities.
- After completion of the practical activities (epidemiological simulation and statistical analysis), there is a significant improvement in the students’ perception of their ability to understand concepts, perform a statistical analysis, and sort the results of the analysis.

## Supporting information

S1 File Perception survey in English and Spanish. (DOCX)

S2 File Survey data and grades data. (XLSX)
