# Supplementary material for: The impact of participatory teaching methods on medical students’ perception of their abilities and knowledge of epidemiology and statistics
Source: PLoS One. 2018 Aug 22;13(8):e0202769. doi: 10.1371/journal.pone.0202769 (PMC6105020; doi:10.1371/journal.pone.0202769)
Supplement: S1 File — (DOCX) [file pone.0202769.s001.docx]

**ANNEX 1: Perception survey.**

The objective of the study is to analyze the effect of the participatory methods on students’ perception of their abilities and knowledge of Epidemiology and Statistics and the importance and usefulness of the course.

Participation is voluntary and anonymous. Data confidentiality is ensured.

Mark with a cross the answer that you consider most appropriate.

Thank you for your cooperation.

|  | Score | | | |
| --- | --- | --- | --- | --- |
| ***Indicate from 1 to 4, where 1 is “strongly disagree” and 4 “strongly agree”, your degree of agreement with the following statements:*** | 1 | 2 | 3 | 4 |
| 1.- Epidemiology and Biostatistics are important for Medicine as a science |  |  |  |  |
| 2.- I believe that the knowledge and skills I have acquired in this course are fundamental for my professional performance in Medicine |  |  |  |  |
| 3.- I understand the relationship between Epidemiology and Biostatistics |  |  |  |  |
| 4.- I understand the main concepts of Epidemiology and Biostatistics |  |  |  |  |
|  |  |  |  |  |
| ***Rate from 1 to 4 your ability to:*** |  |  |  |  |
| 5.- Read and understand scientific articles |  |  |  |  |
| 6.- Perform a basic statistical analysis |  |  |  |  |
| 7.- Sort the data of a statistical analysis |  |  |  |  |
| 8.- Write the results of a statistical analysis |  |  |  |  |
| 9.- Interpret the results of the statistical tests |  |  |  |  |

**ANEXO 1: Encuesta de percepción**

El objetivo del estudio es analizar el efecto de las metodologías activas en la percepción de los estudiantes sus habilidades y conocimientos relacionados con la asignatura y sobre la importancia y utilidad de la misma.

La encuesta es voluntaria y anónima. Se asegura la confidencialidad de los datos.

Señale con una cruz la respuesta que considere más adecuada.

Gracias por su colaboración.

|  | Puntuación | | | |
| --- | --- | --- | --- | --- |
| ***Indique de 1 a 4, (1 indica “totalmente en desacuerdo”; 4 indica “totalmente de acuerdo”), su grado de acuerdo con las siguientes afirmaciones:*** | 1 | 2 | 3 | 4 |
| 1.- La Epidemiología y la Bioestadística son importantes para la Medicina como ciencia |  |  |  |  |
| 2.- Creo que los conocimientos y competencias que he adquirido en esta asignatura son fundamentales para mi desempeño profesional de la Medicina |  |  |  |  |
| 3.- Entiendo la relación entre la Epidemiología y la Bioestadística |  |  |  |  |
| 4.- Entiendo los conceptos principales de Epidemiología y Bioestadística |  |  |  |  |
|  |  |  |  |  |
| ***Valore del 1 al 4 su capacidad para:*** |  |  |  |  |
| 5.- Leer y comprender artículos científicos |  |  |  |  |
| 6.- Hacer un análisis estadístico básico |  |  |  |  |
| 7.- Ordenar los datos de un análisis estadístico |  |  |  |  |
| 8.- Redactar los resultados de un análisis estadístico |  |  |  |  |
| 9.- Interpretar los resultados de las pruebas estadísticas |  |  |  |  |
